# On the Communication–Key Rate Region of Hierarchical Vector Linear Secure Aggregation

**DOI:** 10.3390/e28030352

**Published:** 2026-03-20

**Authors:** Jiawen Lv, Xiang Zhang, Zhou Li

**Affiliations:** 1 Guangxi Key Laboratory of Multimedia Communications and Network Technology, Guangxi University, Nanning 530004, China; lvljw1001@gmail.com; 2 Department of Electrical Engineering and Computer Science, Technical University of Berlin, 10623 Berlin, Germany; xiang.zhang@tu-berlin.de

**Keywords:** hierarchical secure aggregation, vector linear, information-theoretic security, federated learning

## Abstract

Motivated by heterogeneous data distributions and task-dependent aggregation requirements in federated learning, we study information-theoretic secure aggregation of linear functions over a two-hop hierarchical network. The system comprises an aggregation server, an intermediate layer of *U* relays, and UV users, where each relay serves a disjoint cluster of *V* users. Each relay observes all uplink transmissions within its cluster and forwards a coded message to the server. The server is authorized to compute a prescribed linear function *F* of the users’ inputs with zero error, while being prevented from learning any additional information about an unauthorized linear function *G*. Moreover, each relay must obtain no information about any non-trivial linear function $B_{u}$ of the inputs in its own cluster. We define the communication rates on both hops as the number of transmitted symbols per input symbol. By deriving matching information-theoretic converse and achievability bounds, we fully characterize the optimal communication rates and propose an explicit linear coding scheme that achieves the resulting optimal region. Our results demonstrate that hierarchical architectures can attain optimal communication rates while substantially reducing the server-side masking burden, thereby enabling scalable secure aggregation of authorized linear functions.

## 1. Introduction

With the rapid proliferation of machine learning and data analytics applications, massive amounts of data are continuously generated by geographically distributed users and devices. In many practical scenarios, such as healthcare analytics, intelligent transportation, and personalized recommendation systems, these data are highly sensitive. Directly collecting or centrally storing raw user data therefore poses significant privacy risks and regulatory challenges. Secure aggregation has emerged as a fundamental primitive to address this tension, enabling an aggregator to compute desired statistics over distributed data while preventing the disclosure of individual user information.

From an information-theoretic perspective, secure aggregation inherently incurs simultaneous costs in communication efficiency and randomness consumption. A classical starting point is secure aggregation, where each of *K* users holds a private input and transmits a masked message to a server. The server is required to recover the sum of all inputs with zero error while learning no additional information. Prior work [1] has shown that achieving perfect secure inevitably requires a nontrivial amount of randomness, and that reducing communication cost typically increases the required key rate. This communication and randomness relationship has been fully characterized for secure aggregation and several of its variants, establishing randomness as a fundamental resource rather than a mere implementation detail.

As distributed learning systems evolve, secure aggregation alone is insufficient to capture practical requirements. First, the desired computation is often more general than a scalar sum and can be modeled as an arbitrary linear transformation of the users’ data. Second, security requirements are frequently function-specific: while the server is authorized to learn a prescribed linear function *F* of the users’ data, it must be prevented from inferring other sensitive linear functions, denoted by *G*. This motivates the formulation of vector linear secure aggregation, in which the security cost is no longer determined solely by the number of users, but also by the algebraic relationship between the authorized function *F* and the protected functions *G*. In particular, the additional information contained in *G* beyond what is revealed by *F* is quantified by the conditional rank rank(G∣F), which directly determines the minimum amount of randomness required for security.

Most existing information-theoretic results on vector linear secure aggregation focus on single-hop network architectures [2,3], where all users communicate directly with a central server. While such models are analytically convenient, they do not fully reflect the structure of large-scale practical systems. In real deployments, direct communication between a server and a massive number of users can lead to scalability and access limitations. As a result, hierarchical or edge-assisted architectures are widely adopted, in which users first communicate with nearby relays or gateways, and the relays subsequently forward aggregated messages to the server.

Introducing a hierarchical architecture fundamentally changes the secure aggregation problem [4]. Unlike the classical single-hop setting, where only the server’s inference needs to be controlled, a two-hop network creates an additional inference layer: each relay observes all transmissions from users in its cluster and may infer extra intra-cluster linear information unless properly constrained. Meanwhile, the server should recover only a prescribed global linear function of the cluster aggregates and remain ignorant of other unauthorized linear combinations.

Information-theoretic secure aggregation has been extended to a variety of settings, including user dropout [5,6], secure aggregation with user selection [7], designs resilient to user collusion [8,9,10], schemes employing groupwise keys [11,12], secure aggregation with oblivious servers [13], secure aggregation under unreliable communication [14], and hierarchical secure aggregation [15,16,17,18,19]. Other related works on secure aggregation from different perspectives can be found in [20,21,22,23].

However, existing works do not characterize the vector linear two-hop hierarchical setting within a unified information-theoretic framework, where relay-side protection against unauthorized intra-cluster linear inference and server-side recovery of only a prescribed global function must be enforced simultaneously. Our contribution is not only to unify hierarchical secure aggregation and vector linear secure aggregation within a single information-theoretic model, but also to show that the resulting two-hop formulation exhibits genuinely coupled relay-side and server-side security constraints, leading to a new optimal key-rate characterization and requiring a joint algebraic coding design.

To further clarify the distinction from prior single-hop vector linear secure aggregation schemes, Table 1 summarizes the main differences between those formulations and the proposed two-hop hierarchical setting.

In this work, we study information-theoretic vector linear secure aggregation over a two-hop hierarchical network consisting of *U* relays, each serving a disjoint cluster of *V* users. The server is required to recover, with zero error, a prescribed linear function *F* of the cluster aggregates while learning no additional information about an unauthorized linear function *G*. At the same time, each relay may assist local aggregation but must remain ignorant of the unauthorized intra-cluster linear functions characterized by $B_{u}$ within its own cluster. Our goal is to completely characterize the fundamental communication and randomness limits of this problem.

We prove that, in the unified hierarchical vector linear secure aggregation model, the communication optimality remains unchanged compared with the single-hop setting: the first-hop rate still satisfies $R_{X}=1$, and the second-hop rate can still achieve $R_{Y}=1$ even after introducing an additional relay layer. However, the minimum source key rate changes from depending only on rank(G∣F) in the single-hop model to being jointly determined by the relay-side intra-cluster protection requirement $K_{u}$ and the server-side protection constraint rank(G∣F). This shows that, although the hierarchical structure does not increase the communication cost, it introduces a coupling between relay-side security and server-side function security in the key design.

From a technical standpoint, establishing the fundamental limits is challenging because both the converse and the achievability must simultaneously account for relay-side intra-cluster secrecy and server-side function authorization. In particular, the converse requires a joint information-theoretic argument for the two levels of security, while the achievability calls for a unified linear coding design that preserves local privacy, enables authorized global recovery, and maintains optimal communication rates over both hops.

We further provide an explicit linear coding scheme that achieves these fundamental limits.

## 2. Problem Statement

**Figure 1:**
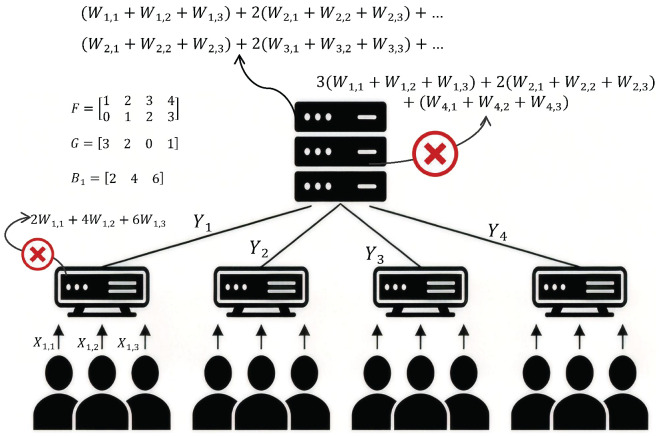
Illustration of hierarchical secure linear aggregation. The aggregation server is permitted to compute information about the linear function *F*, but is not permitted to compute any information about the linear function *G*. In addition, each relay is required to be unable to compute any intra-cluster information about the linear function $B_{u}$ (intra-cluster privacy constraint).

Consider a three-layer hierarchical secure aggregation system consisting of an aggregation server, an intermediate layer of U≥2 relays, and a bottom layer of UV users. The network operates over two hops, where the server communicates with all relays and each relay serves a disjoint cluster of exactly *V* users, as illustrated in Figure 1. All communication links are assumed to be error-free. We consider a static system model with fixed cluster size, where no user dropout occurs during the protocol. We further assume that no collusion takes place among users, relays, and the server, and that all entities follow the prescribed protocol without adversarial or Byzantine behavior. The *v*-th user associated with the *u*-th relay is indexed by (u,v)∈[U]×[V]. Each user (u,v) holds a private input $W_{u,v}$ over a finite field $F_{q}$ with entropy $H(W_{u,v})=L$ measured in *q*-ary units, and the inputs are assumed to be independent and uniformly distributed across users. In addition, each user (u,v) is equipped with a local key variable $Z_{u,v}$, satisfying $H\left(Z_{u,v}\right)=L_{Z}$. The collection of individual keys $Z_{\left[U]\times [V\right]}≜{\left\{Z_{u,v}\right\}}_{u\in [U],\;v\in [V]}$ is deterministically generated from a common source key variable $Z_{\Sigma }$, where $H\left(Z_{\Sigma }\right)=L_{Z_{\Sigma }}$. The source key $Z_{\Sigma }$ is generated and securely distributed by a trusted third-party entity. The key variables $Z_{\left[U]\times [V\right]}$ are statistically independent of the user inputs $W_{\left[U]\times [V\right]}≜{\left\{W_{u,v}\right\}}_{u\in [U],\;v\in [V]}$.

(1) $$\begin{array}{cc} \; & H\left(Z_{\left[U]\times [V\right]},W_{\left[U]\times [V\right]}\right)=H\left(Z_{\left[U]\times [V\right]}\right)+\sum_{u\in [U],v\in [V]}H\left(W_{u,v}\right), \end{array}$$

(2) $$\begin{array}{cc} \; & H\;\left(Z_{\left[U]\times [V\right]}|Z_{\Sigma }\right)=0. \end{array}$$

The system adopts a two-hop communication protocol. In the first hop, User (u,v) transmits a message $X_{u,v}$ to its associated relay. The message $X_{u,v}$ is generated as a function of the local input $W_{u,v}$ and the local key $Z_{u,v}$, and consists of $L_{X}$ symbols. In the second hop, relay *u* transmits a message $Y_{u}$ to the aggregation server. The message $Y_{u}$ consists of $L_{Y}$ symbols and is generated as a function of the received messages ${\left\{X_{u,v}\right\}}_{v\in [V]}$ from Users in cluster *u*.

(3) $$\begin{array}{ccc} & & H\;\left(X_{u,v}|W_{u,v},Z_{u,v}\right)=0,\;\forall \left(u,v\right)\in \left[U\right]\times \left[V\right], \end{array}$$

(4) $$\begin{array}{ccc} & & H\;\left(Y_{u}|{\left\{X_{u,v}\right\}}_{v\in [V]}\right)=0,\;\forall u\in \left[U\right]. \end{array}$$

We define the cluster aggregate at relay *u* as the sum of the users’ inputs within cluster *u*, i.e.,

(5) $$\begin{array}{c} S_{u}≜\sum_{v\in [V]}W_{u,v},\;u\in \left[U\right]. \end{array}$$

In general, the relay message $Y_{u}$ can be an arbitrary function of the received messages ${\left\{X_{u,v}\right\}}_{v\in [V]}$. Specifically, in this work, we restrict attention to schemes in which the relay message $Y_{u}$ is a deterministic function of the cluster aggregate $S_{u}$ and the local keys ${\left\{Z_{u,v}\right\}}_{v\in [V]}$, i.e.,

(6) $$\begin{array}{c} H\;\left(Y_{u}|S_{u},{\left\{Z_{u,v}\right\}}_{v\in [V]}\right)=0,\;\forall u\in \left[U\right]. \end{array}$$

From the relay messages, the aggregation server aims to recover an authorized linear function *F* while revealing no information about an unauthorized linear function *G* in the information-theoretic sense. Define $S≜\left[S_{1};\ldots ;S_{U}\right]\in F_{q}^{U\times L}.$ The functions *F* and *G* are given by

(7) $$\begin{array}{c} F=FS\in F_{q}^{M\times L},\;\;\;G=GS\in F_{q}^{N\times L}, \end{array}$$

where $F\in F_{q}^{M\times U}$ and $G\in F_{q}^{N\times U}$ are assumed to have full row rank, i.e., M=rank(F) and N=rank(G), without loss of generality.

To prevent trivial cases, we assume that F contains no zero columns. A zero column associated with $S_{u}$ would indicate that $S_{u}$ has no effect on the computation of *F* and could thus be excluded without affecting the problem.

From the relay’s messages, the server should be able to recover the desired linear function *F*, i.e.,

(8) $$\begin{array}{c} \left[\mathrm{Correctness}\right]\;H\;\left(F\;|\;{\left\{Y_{u}\right\}}_{u\in [U]}\right)=0. \end{array}$$

The security constraints require that each relay should not gain any information about any unauthorized linear function $B_{u}$ from the messages transmitted by its associated users. Specifically, let $W_{u}≜\left[W_{u,1};\ldots ;W_{u,V}\right]\in F_{q}^{V\times L},$ and define the unauthorized function

(9) $$B_{u}=B_{u}W_{u}\in F_{q}^{K_{u}\times L},$$

where $B_{u}\in F_{q}^{K_{u}\times V}$ is assumed to have full row rank without loss of generality, i.e., $K_{u}=\mathrm{rank}\left(B_{u}\right),u\in \left[U\right]$. The relay security constraint can then be expressed as

(10) $$\begin{array}{c} I\;\left(B_{u};\;{\left\{X_{u,v}\right\}}_{v\in [V]}\right)=0,\;\forall u\in \left[U\right]. \end{array}$$

In addition, the server must not learn any information about the unauthorized function *G* beyond what is already contained in the authorized function *F*. This server security constraint is written as

(11) $$\begin{array}{c} I\;\left(G;{\left\{Y_{u}\right\}}_{u\in [U]}|F\right)=0. \end{array}$$

The communication rates $R_{X}$ and $R_{Y}$ are defined as the numbers of symbols in each transmitted message $X_{u,v}$ and $Y_{u}$, respectively, normalized by the input length *L*. Similarly, the source key rate $R_{Z_{\Sigma }}$ represents the number of symbols in the key variable $Z_{\Sigma }$ per input symbol. Formally,

(12) $$\begin{array}{c} R_{X}≜\frac{L_{X}}{L},\;R_{Y}≜\frac{L_{Y}}{L},\;R_{Z_{\Sigma }}≜\frac{L_{Z_{\Sigma }}}{L}. \end{array}$$

A rate tuple $\left(R_{X},R_{Y},R_{Z_{\Sigma }}\right)$ is said to be achievable if there exists a secure aggregation scheme, specified by the key variable $Z_{\Sigma }$, and the transmitted messages ${\left\{X_{u,v}\right\}}_{\left(u,v)\in [U]\times [V\right]}$ and ${\left\{Y_{u}\right\}}_{u\in [U]}$, satisfying (3) and (4), such that the communication and key rates are $\left(R_{X},R_{Y},R_{Z_{\Sigma }}\right)$ and the correctness constraint (8) together with the security constraints (10) and (11) are all met. The optimal rate region $R^{*}$ is defined as the closure of the set of all achievable rate tuples.

## 3. Main Results

In this section, we present the main results of this work. The optimal vector linear communication and key rate region for the hierarchical vector linear secure aggregation problem is characterized in Theorem 1.

**Theorem** **1.** *For the hierarchical vector linear secure aggregation problem described above, the optimal vector linear communication and key rate region is*

(13) $$\begin{array}{cc} R^{*}= & \left\{\left(R_{X},R_{Y},R_{Z_{\Sigma }}\right)|R_{X}\geq 1,\;R_{Y}\geq 1,\;R_{Z_{\Sigma }}\geq \max\left\{\max_{u\in [U]}K_{u},\;\mathrm{rank}\left(G|F\right)\right\}\right\}, \end{array}$$

*where*

(14) $$\mathrm{rank}\left(G|F\right)=\mathrm{rank}\;\left([F;G]\right)-\mathrm{rank}\left(F\right).$$

*Moreover, the converse holds under the stated model, and the above region is achievable by a vector linear coding scheme over sufficiently large finite fields.
*

## 4. Motivating Example (*U* = 4, *V* = 3, *M* = 2, *N* = 1)

Prior to describing the general achievability scheme in Theorem 1, we introduce a representative example to convey the key principles behind the proposed hierarchical vector linear secure aggregation problem. These examples serve to build intuition for the design, after which the complete construction is presented.

Consider a two-hop hierarchical system with U=4 relays and V=3 users per cluster. In the first hop, each relay aggregates the messages from users in its corresponding cluster while being prevented from learning any information about the linear function $B_{u}W$, where $W≜{\left[W_{u,1},W_{u,2},W_{u,3}\right]}^{T}\in F_{7}^{3\times 1},$ and $B_{u}$ is specified as follows.

(15) $$\begin{array}{cc} B_{1} & =\left[\begin{array}{ccc} 2 & 4 & 6 \end{array}\right],\;B_{2}=\left[\begin{array}{ccc} 3 & 5 & 1 \end{array}\right],\\ B_{3} & =\left[\begin{array}{ccc} 1 & 3 & 2\\ 3 & 6 & 1 \end{array}\right]\overset{\;\mathrm{row}/\mathrm{column}\;\mathrm{operations}}{\to }\left[\begin{array}{ccc} 1 & 0 & 4\\ 0 & 1 & 4 \end{array}\right],\\ B_{4} & =\left[\begin{array}{ccc} 1 & 2 & 3\\ 4 & 5 & 6\\ 5 & 0 & 2 \end{array}\right]\overset{\;\mathrm{row}/\mathrm{column}\;\mathrm{operations}}{\to }\left[\begin{array}{ccc} 1 & 0 & 6\\ 0 & 1 & 2\\ 0 & 0 & 0 \end{array}\right]. \end{array}$$

In the second hop, the server aims to recover FS from the messages uploaded by all relays with zero error, where $S≜{\left[S_{1},S_{2},S_{3},S_{4}\right]}^{T}\in F_{7}^{4\times 1},\;S_{u}=\sum_{v\in [3]}W_{u,v},\;u\in \left[4\right].$ Moreover, the server must not obtain any additional information about GS beyond what is implied by FS.

(16) $$F=\left[\begin{array}{cccc} 1 & 2 & 3 & 4\\ 0 & 1 & 2 & 3 \end{array}\right],\;G=\left[\begin{array}{cccc} 3 & 2 & 0 & 1 \end{array}\right].$$

(17) $$F=\left[\begin{array}{cccc} 1 & 2 & 3 & 4\\ 0 & 1 & 2 & 3 \end{array}\right]\;\overset{\mathrm{row}/\mathrm{column}\;\mathrm{operations}}{\to }\;\left[\begin{array}{cccc} 1 & 0 & 6 & 5\\ 0 & 1 & 2 & 3 \end{array}\right].$$

Consequently, we have

(18) $$\begin{array}{cc} FS\; & =\left[\begin{array}{c} S_{1}+2S_{2}+3S_{3}+4S_{4}\\ S_{2}+2S_{3}+3S_{4} \end{array}\right]\\ \; & =\left[\begin{array}{c} \sum_{v\in [3]}W_{1,v}+2\sum_{v\in [3]}W_{2,v}+3\sum_{v\in [3]}W_{3,v}+4\sum_{v\in [3]}W_{4,v}\\ \sum_{v\in [3]}W_{2,v}+2\sum_{v\in [3]}W_{3,v}+3\sum_{v\in [3]}W_{4,v} \end{array}\right], \end{array}$$

(19) $$\begin{array}{cc} GS\; & =3S_{1}+2S_{2}+S_{4}\\ \; & =3\sum_{v\in [3]}W_{1,v}+2\sum_{v\in [3]}W_{2,v}+\sum_{v\in [3]}W_{4,v}. \end{array}$$

where GS is a scalar linear combination of the components of S.

Consider the second hop and set L=1. Based on (17), suppose we have two independent and uniformly distributed noise variables $T_{1},T_{2}$ over $F_{7}$. Then we have

(20) $$\begin{array}{cc} Y_{1}\; & =S_{1}-6T_{1}-5T_{2}=W_{1,1}+W_{1,2}+W_{1,3}-6T_{1}-5T_{2},\\ Y_{2}\; & =S_{2}-2T_{1}-3T_{2}=W_{2,1}+W_{2,2}+W_{2,3}-2T_{1}-3T_{2},\\ Y_{3}\; & =S_{3}+T_{1}=W_{3,1}+W_{3,2}+W_{3,3}+T_{1},\\ Y_{4}\; & =S_{4}+T_{2}=W_{4,1}+W_{4,2}+W_{4,3}+T_{2}. \end{array}$$

For the server security constraint, only 1 key symbol is required. It turns out that $T_{1}$ and $T_{2}$ need not be independent; introducing correlation between them in the next step is the most technical part of the proof.

We then seek a 1×2 matrix $Q\in F_{q}^{1\times 2}$ that characterizes the correlation between $\left(T_{1},T_{2}\right)$, such that

(21) FG00Qhasfullrank4.

Note that such a matrix Q exists since rank([F;G])=3. Consequently, there always exists a nonzero 1×2 vector Q that completes (21) to full rank. Any valid choice of Q suffices for our purpose.

We then compute the right null space of Q, denoted by $Q^{⊥}\in F_{q}^{2\times 1}$, which satisfies

(22) $$Q=\left[\begin{array}{cc} 1 & 3 \end{array}\right],\;Q^{⊥}=\left[\begin{array}{c} 4\\ 1 \end{array}\right].$$

Then the key symbols $T_{1}$ and $T_{2}$ can be generated from a single uniformly distributed key symbol $P_{1}$ by precoding with $Q^{⊥}$,

(23) $$\left[\begin{array}{c} T_{1}\\ T_{2} \end{array}\right]=Q^{⊥}P_{1}=\left[\begin{array}{c} 4P_{1}\\ P_{1} \end{array}\right].$$

We may write out the final message assignment using the single key symbol $P_{1}$:

(24) $$\begin{array}{cc} Y_{1} & =W_{1,1}+W_{1,2}+W_{1,3}-P_{1},\;\;\;\;\;\;\;\;\;\;\;\;\;\;Y_{2}=W_{2,1}+W_{2,2}+W_{2,3}-4P_{1},\\ Y_{3} & =W_{3,1}+W_{3,2}+W_{3,3}+4P_{1},\;\;\;\;\;\;\;\;\;\;\;\;Y_{4}=W_{4,1}+W_{4,2}+W_{4,3}+P_{1}. \end{array}$$

The signal observed at relay *u* can be expressed as

(25) $$Y_{u}≜S_{u}+Z_{u}^{Y},$$

where $Z_{u}^{Y}$ denotes the key component embedded in $Y_{u}$.

In *Example 1*, this decomposition admits an explicit representation:

(26) $$\left[\begin{array}{c} Z_{1}^{Y}\\ Z_{2}^{Y}\\ Z_{3}^{Y}\\ Z_{4}^{Y} \end{array}\right]=\left[\begin{array}{c} -1\\ -4\\ 4\\ 1 \end{array}\right]P_{1}.$$

Next, we investigate the security of relay 4 under the proposed key assignment. Since $\mathrm{rank}(B_{4})=2$, relay 4 requires at least $K_{4}=2$ independent keys, denoted by $N_{1}$ and $N_{2}$. There exists a matrix $A\in F_{q}^{1\times 2}$ such that

$$P_{1}=AN=a_{11}N_{1}+a_{12}N_{2}.$$

Since the coefficients of A can be any nonzero values in $F_{q}$, for simplicity we set $a_{11}=a_{12}=1$, yielding

$$P_{1}=N_{1}+N_{2}.$$

Therefore, the relay messages can be written as

(27) $$\begin{array}{cccc} Y_{1} & =S_{1}-\left(N_{1}+N_{2}\right), & Y_{2} & =S_{2}-4\left(N_{1}+N_{2}\right),\\ Y_{3} & =S_{3}+4\left(N_{1}+N_{2}\right), & Y_{4} & =S_{4}+\left(N_{1}+N_{2}\right). \end{array}$$

To prevent relay 4 from obtaining any information regarding the linear function $B_{4}X$, we require

(28) $$B_{4}\left[\begin{array}{c} X_{1}\\ X_{2}\\ X_{3} \end{array}\right]=B_{4}\left[\begin{array}{c} W_{4,1}\\ W_{4,2}\\ W_{4,3} \end{array}\right]+\underset{\neq 0}{\underset{︸}{B_{4}\left[\begin{array}{c} Z_{4,1}\\ Z_{4,2}\\ Z_{4,3} \end{array}\right]}}.$$

Specifically, the interference term is constructed using the keys as

(29) $$B_{4}\left[\begin{array}{c} Z_{4,1}\\ Z_{4,2}\\ Z_{4,3} \end{array}\right]=\underset{\neq 0}{\underset{︸}{B_{4}R_{4}}}\left[\begin{array}{c} N_{1}\\ N_{2} \end{array}\right],\;R_{4}\in F_{q}^{3\times 2}.$$

Equivalently, we can write

(30) $$\left[\begin{array}{c} Z_{4,1}\\ Z_{4,2}\\ Z_{4,3} \end{array}\right]=R_{4}\left[\begin{array}{c} N_{1}\\ N_{2} \end{array}\right],\;R_{4}\in F_{q}^{3\times 2}.$$

To ensure that the noise term fully masks the signal space and cannot be nullified via linear projection, the product $B_{4}R_{4}$ must have full rank, i.e.,

$$\mathrm{rank}(B_{4}R_{4})=2.$$

Specifically, we construct the first two rows of $R_{4}$ as a full-rank block to ensure linear independence, and utilize the last row to satisfy the aggregation coefficient constraints. Consequently, as shown in (26), the aggregated interference term $Z_{4}^{Y}$ yields the summation of the keys:

(31) $$Z_{4}^{Y}=1\cdot P_{1}=\left[\begin{array}{cc} 1 & 1 \end{array}\right]\left[\begin{array}{c} N_{1}\\ N_{2} \end{array}\right]=N_{1}+N_{2}.$$

The corresponding precoding matrix is

(32) $$R_{4}=\left[\begin{array}{cc} -1 & 0\\ 0 & -1\\ 2 & 2 \end{array}\right]\in F_{q}^{3\times 2}.$$

The left null space of $R_{4}$ is

(33) $$R_{4}^{⊥}=\left[\begin{array}{ccc} 2 & 2 & 1 \end{array}\right]\in F_{q}^{1\times 3},\;\mathrm{with}\;R_{4}^{⊥}R_{4}=0.$$

This construction ensures that $R_{4}$ has full column rank, $\mathrm{rank}(R_{4})=2$, satisfying the required rank condition.

Similarly, for relay 1, since $\mathrm{rank}(B_{1})=1$, it requires only one independent key, namely $\left(N_{1}+N_{2}\right)$. To satisfy the condition $\mathrm{rank}(B_{1}R_{1})=1$, we construct $R_{1}$ as follows:

$$Z_{1}^{Y}=-1\cdot P_{1}=-1\;\left[\;N_{1}+N_{2}\;\right].$$

(34) $$R_{1}=\left[\begin{array}{c} 1\\ 1\\ -3 \end{array}\right].$$

Consequently, the left null space of $R_{1}$ is given by

(35) $$R_{1}^{⊥}=\left[\begin{array}{ccc} 6 & 1 & 0\\ 3 & 0 & 1 \end{array}\right],\;\mathrm{satisfying}\;R_{1}^{⊥}R_{1}=0.$$

Similarly, for the other relays 2 and 3, we construct $R_{2}$ and $R_{3}$, from which the individual user keys are obtained as follows:

(36) $$\begin{array}{cccccc} Z_{1,1} & =N_{1}+N_{2}, & \;Z_{1,2} & =N_{1}+N_{2}, & \;Z_{1,3} & =-3N_{1}-3N_{2},\\ Z_{2,1} & =N_{1}+N_{2}, & \;Z_{2,2} & =N_{1}+N_{2}, & \;Z_{2,3} & =-6N_{1}-6N_{2},\\ Z_{3,1} & =N_{1}, & \;Z_{3,2} & =N_{2}, & \;Z_{3,3} & =3N_{1}+3N_{2},\\ Z_{4,1} & =-N_{1}, & \;Z_{4,2} & =-N_{2}, & \;Z_{4,3} & =2N_{1}+2N_{2}. \end{array}$$

Since $L_{X}=L_{Y}=1$ and $L_{Z_{\Sigma }}=2$, the resulting rates are

$$R_{X}=R_{Y}=1,\;R_{Z_{\Sigma }}=2,$$

which match the converse bound established in Theorem 1.

**Correctness:** From the received signals $Y_{1},Y_{2},Y_{3},Y_{4}$, the server applies the linear transform F and successfully recovers
  F=FS
  with zero error.
**Relay security:** From the transformation in (15), it follows that
  $$K_{u}=1\;\mathrm{for}\;u\in \left\{1,2\right\},\;K_{u}=2\;\mathrm{for}\;u\in \left\{3,4\right\}.$$

Since relays whose $B_{u}$ have the same rank require the same total number of independent masking key symbols, it suffices to establish the security proof for relays 1 and 4; the cases of relays 2 and 3 follow by analogous arguments.

Consider relay 4, for example:

(37) $$\begin{array}{cc} \; & I(\left\{B_{4}\right\};{\left\{X_{4,v}\right\}}_{v\in [3]}) \end{array}$$

(38) $$\begin{array}{cc} \; & =H\left(X_{4,1},X_{4,2},X_{4,3}\right)-H\;\left(X_{4,1},X_{4,2},X_{4,3}|B_{4}\right) \end{array}$$

(39) $$\begin{array}{cc} \; & \leq 3-H\;\left(X_{4,1},X_{4,2},X_{4,3},R_{4}^{⊥}\left[X_{4,1},X_{4,2},X_{4,3}\right]|B_{4}\right) \end{array}$$

(40) $$\begin{array}{cc} \; & =3-H\;\left(X_{4,1},X_{4,2},X_{4,3},R_{4}^{⊥}\left[W_{4,1},W_{4,2},W_{4,3}\right]|B_{4}\right) \end{array}$$

(41) $$\begin{array}{cc} \; & =3-H\;\left(R_{4}^{⊥}\left[W_{4,1},W_{4,2},W_{4,3}\right]|B_{4}\right)-H\;\left(X_{4,1},X_{4,2},X_{4,3}|R_{4}^{⊥}\left[W_{4,1},W_{4,2},W_{4,3}\right],B_{4}\right) \end{array}$$

(42) $$\begin{array}{cc} \; & =3-\mathrm{rank}\left(R_{4}^{⊥}\right)-H\left(N_{1},N_{2}\right) \end{array}$$

(43) $$\begin{array}{cc} \; & =3-1-2=0. \end{array}$$

In (40), we adopt a zero-forcing strategy by constructing the precoding matrix $R_{4}^{⊥}$ so that the key components are perfectly eliminated in its left null space, i.e., $R_{4}^{⊥}R_{4}=0.$ In (42), the second term holds because $R_{4}^{⊥}R_{4}=0,$ and $R_{4}^{⊥}\left[W_{4,1},W_{4,2},W_{4,3}\right]$ is independent of $B_{4}$. Moreover, the matrix formed by $R_{4}^{⊥}\left[W_{4,1},W_{4,2},W_{4,3}\right]$ and $B_{4}$ has full rank, and hence is invertible with respect to $W_{4,1},W_{4,2},W_{4,3}$.

Consider relay 1, for example:

(44) $$\begin{array}{cc} \; & I(\left\{B_{1}\right\};{\left\{X_{1,v}\right\}}_{v\in [3]})\\ \; & =H\left(X_{1,1},X_{1,2},X_{1,3}\right)-H\left(X_{1,1},X_{1,2},X_{1,3}|B_{1}\right) \end{array}$$

(45) $$\begin{array}{cc} \; & \leq 3-H\left(X_{1,1},X_{1,2},X_{1,3},R_{1}^{⊥}\left[X_{1,1},X_{1,2},X_{1,3}\right]|B_{1}\right) \end{array}$$

(46) $$\begin{array}{cc} \; & =3-H\left(X_{1,1},X_{1,2},X_{1,3},R_{1}^{⊥}\left[W_{1,1},W_{1,2},W_{1,3}\right]|B_{1}\right) \end{array}$$

(47) $$\begin{array}{cc} \; & \overset{\left(35\right)}{=}3-H\left(R_{1}^{⊥}\left[W_{1,1},W_{1,2},W_{1,3}\right]|B_{1}\right)-H\left(X_{1,1},X_{1,2},X_{1,3}|R_{1}^{⊥}\left[W_{1,1},W_{1,2},W_{1,3}\right],B_{1}\right) \end{array}$$

(48) $$\begin{array}{cc} \; & =3-\mathrm{rank}\left(R_{1}^{⊥}\right)-H\left(N_{1}+N_{2}\right) \end{array}$$

(49) $$\begin{array}{cc} \; & =3-2-1=0. \end{array}$$

In (46), we adopt a zero-forcing strategy by constructing the precoding matrix $R_{1}^{⊥}$ so that the key components are perfectly eliminated in its left null space, i.e., $R_{1}^{⊥}R_{1}=0.$ In (48), the second term holds because $R_{1}^{⊥}R_{1}=0,$ and $R_{1}^{⊥}\left[W_{1,1},W_{1,2},W_{1,3}\right]$ is independent of $B_{1}$. Moreover, the matrix formed by $R_{1}^{⊥}\left[W_{1,1},W_{1,2},W_{1,3}\right]$ and $B_{1}$ has full rank, and hence is invertible with respect to $W_{1,1},W_{1,2},W_{1,3}$.

We now proceed to present the security proof for the server.

$$\begin{array}{cc} \; & I(G;Y_{1},Y_{2},Y_{3},Y_{4}|F) \end{array}$$

(50) $$\begin{array}{cc} \; & =H\left(Y_{1},Y_{2},Y_{3},Y_{4}|F\right)-H\left(Y_{1},Y_{2},Y_{3},Y_{4}|G,F\right) \end{array}$$

(51) $$\begin{array}{cc} \; & =\left[H\left(Y_{1},Y_{2},Y_{3},Y_{4},F\right)-H\left(F\right)\right]-H\left(Y_{1},Y_{2},Y_{3},Y_{4},Q\left[Y_{3},Y_{4}\right]|G,F\right) \end{array}$$

(52) $$\begin{array}{cc} \; & =\left[H\left(Y_{1},Y_{2},Y_{3},Y_{4},F\right)-H\left(F\right)\right]-H\left(Y_{1},Y_{2},Y_{3},Y_{4},Q\left[S_{3},S_{4}\right]|G,F\right) \end{array}$$

(53) $$\begin{array}{cc} \; & \leq \left(4-2\right)-H\left(Q\left[S_{3},S_{4}\right]|G,F\right)-H\left(Y_{1},Y_{2},Y_{3},Y_{4}|Q\left[S_{3},S_{4}\right],G,F\right) \end{array}$$

(54) $$\begin{array}{cc} \; & =2-1-H(P_{1}) \end{array}$$

(55) $$\begin{array}{cc} \; & =2-1-H(N_{1}+N_{2})=2-1-1=0, \end{array}$$

where (52) follows from the orthogonality $QQ^{⊥}=0$, which implies that the noise components precoded by $Q^{⊥}$ are completely eliminated (zero-forced) when left-multiplied by Q, cf. (22) and (23). Concerning (54), we leverage the full-rank properties of $Q[S_{3};S_{4}]$, GS, and FS, which ensure the unique solvability of $S_{1},\ldots ,S_{4}$ (see (21)).

## 5. General Achievability Proof of Theorem 1

### 5.1. Conceptual Overview of the Construction

Before introducing the detailed algebraic construction, we briefly explain the guiding idea of the scheme. Transforming F into systematic form makes its right null space explicit. For

$$F=\left[\begin{array}{cc} I_{M} & {\tilde{F}}_{M\times (U-M)} \end{array}\right],\;F\left[\begin{array}{c} -{\tilde{F}}_{M\times (U-M)}\\ I_{U-M} \end{array}\right]=0.$$

Therefore, the systematic form of F explicitly characterizes all key-injection directions that preserve the authorized function FS, thereby ensuring correctness. The role of Q is then to further restrict key injection to a smaller effective subspace within Null(F), containing only the minimum number of directions needed to perfectly hide the unauthorized function GS conditioned on FS.

At this stage, the aggregate noise $Z_{u}^{Y}$ at relay *u* has already been specified by the server-side design. It remains to assign user-level keys such that their aggregate equals $Z_{u}^{Y}$, while satisfying the relay-side privacy and aggregation constraints.

For the relay-side privacy requirement, writing $B_{u}$ in systematic form enables a compatible canonical parametrization of the key-assignment matrix $R_{u}$, under which the full-rank condition on $B_{u}R_{u}$ is reduced to an invertibility constraint on $L_{u}$, as shown in (68)–(70); such a constraint is always feasible over a sufficiently large finite field. Meanwhile, enforcing $1_{V}^{⊤}R_{u}=e_{1}^{⊤}$ in (73), equivalently (74), ensures that the user-level noise aggregates precisely into the prescribed cluster-level noise $Z_{u}^{Y}$. Meanwhile, $R_{u}^{⊥}$ is not part of the construction of $R_{u}$ itself, but is introduced for the relay privacy proof, where its left-null-space property is used to zero-force the injected keys.

### 5.2. General Construction

We now present the general achievability scheme for the two-hop hierarchical vector linear secure aggregation problem. Building on the intuition provided by the motivating example, we construct a unified linear coding scheme and show that it simultaneously guarantees correctness, relay-side security, and server-side function authorization while achieving the claimed communication and key rates.

Given that F has full row rank, we may, without loss of generality, transform it into the following systematic form via column permutations and invertible row operations:

(56) $$F=\left[\begin{array}{cc} I_{M} & {\tilde{F}}_{M\times (U-M)} \end{array}\right],$$

where $I_{M}$ denotes the M×M identity matrix, and $\tilde{F}\in F_{q}^{M\times (U-M)}$ represents the remaining submatrix.

The rows of Q are constructed to be linearly independent of the row space of [F;G], thereby completing a basis of $F_{q}^{U}$. Specifically, we select any U−rank([F;G]) row vectors that are linearly independent of [F;G], and then use the identity submatrix in the first *M* columns of F to linearly eliminate their first *M* components, yielding Q.

The resulting (U−rank([F;G]))×(U−M) matrix Q satisfies

(57) $$\left[\begin{array}{cc} F\\ G\\ 0_{(U-\mathrm{rank}([F;G]))\times M} & Q \end{array}\right]\;\mathrm{has}\;\mathrm{full}\;\mathrm{rank}\;U,$$

which guarantees that the row spaces of F, G, and Q together span the entire ambient space $F_{q}^{U}$.

Intuitively, Q selects and compresses the residual degrees of freedom that are linearly independent of the row space of F into lower-dimensional injection directions. This enables key injection without affecting the F-related structure and avoids using degrees of freedom observable through G. By reordering the columns if necessary, Q can be written in the following block form:

(58) $$\begin{array}{cc} Q & =\left[\begin{array}{cc} I_{U-\mathrm{rank}([F;G])} & \tilde{Q} \end{array}\right],\\ Q^{⊥} & ={\left[\begin{array}{c} -\tilde{Q}\\ I_{\mathrm{rank}([F;G])-M} \end{array}\right]}_{\left(U-M)\times (\mathrm{rank}([F;G])-M\right)},\\ QQ^{⊥} & =0. \end{array}$$

We are now ready to describe the secure aggregation protocol. Set L=1 and define $L_{Z_{\Sigma }}≜\max\left\{\max_{u\in [U]}K_{u},\;\mathrm{rank}\left(G|F\right)\right\}.$ Let $N≜[N_{1};\ldots ;N_{L_{Z_{\Sigma }}}]$ consist of mutually independent and uniformly distributed key symbols.

We generate the key vector $P=AN,\;P\in F_{q}^{\mathrm{rank}(G|F)\times 1},$ where $A\in F_{q}^{\mathrm{rank}\left(G|F\right)\times L_{Z_{\Sigma }}}$ is chosen to be full row rank over $F_{q}$. The injected key symbols are then defined as

(59) $$T≜\left[T_{1};\ldots ;T_{U-M}\right]=Q^{⊥}P=Q^{⊥}AN.$$

The transmitted symbols are constructed as

(60) $$\begin{array}{cc} \left[Y_{1};\ldots ;Y_{M}\right] & =\left[S_{1};\ldots ;S_{M}\right]-\tilde{F}\;\left[T_{1};\ldots ;T_{U-M}\right],\\ Y_{M+1};\ldots ;Y_{U}] & =\left[S_{M+1};\ldots ;S_{U}\right]+\left[T_{1};\ldots ;T_{U-M}\right]. \end{array}$$

Based on (60), the key design for each relay $Z_{u}^{Y}$ is constructed as follows:

(61) $$\begin{array}{cc} T≜[T_{1};\ldots ;T_{U-M}] & =Q^{⊥}P=Q^{⊥}AN\\ {}[Z_{1}^{Y};\ldots ;Z_{M}^{Y}] & =-\tilde{F}\;Q^{⊥}P=-\tilde{F}Q^{⊥}AN\\ {}[Z_{M+1}^{Y};\ldots ;Z_{U}^{Y}] & =\left[T_{1};\ldots ;T_{U-M}\right]=Q^{⊥}P=Q^{⊥}AN. \end{array}$$

(62) $$\left[\begin{array}{c} Z_{1}^{Y}\\ \vdots \\ Z_{U}^{Y} \end{array}\right]=\underset{≜\;\Phi \in F_{q}^{U\times L_{Z_{\Sigma }}}}{\underset{︸}{\left[\begin{array}{c} -\tilde{F}\\ I_{U-M} \end{array}\right]Q^{⊥}A}}N.$$

Let $\beta_{u}^{⊤}\in F_{q}^{1\times L_{Z_{\Sigma }}}$ denote the *u*-th row of Φ.

Thus, for each u∈[U], we have

(63) $$Z_{u}^{Y}=\beta_{u}^{⊤}N.$$

Next, we extend the achievability to the general relay case. Without loss of generality, let $B_{u}$ be represented in its systematic form:

(64) $$B_{u}=\left[\begin{array}{cc} I_{K_{u}} & {\tilde{B}}_{K_{u}\times \left(V-K_{u}\right)} \end{array}\right],$$

as any $B_{u}$ can be transformed into this form via column permutations and invertible row operations.

Let $N^{\left(u\right)}≜D_{u}N$, where $D_{u}\in F_{q}^{K_{u}\times L_{Z_{\Sigma }}}$ is a full-row-rank matrix that maps the global key vector to a relay-specific key vector ($\mathrm{rank}\left(D_{u}\right)=K_{u}$).

At this stage, the noise $Z_{u}^{Y}$ has already been fixed by the server-side design. Moreover, $D_{u}$ is chosen such that

(65) $$e_{1}^{⊤}D_{u}=\beta_{u}^{⊤},$$

where $e_{1}={\left[1,0,\ldots ,0\right]}^{⊤}\in F_{q}^{K_{u}}$ selects the first row of $D_{u}$. Hence,

(66) $$e_{1}^{⊤}D_{u}N=\beta_{u}^{⊤}N,$$

so that the first component of $N^{\left(u\right)}$ coincides with the prescribed cluster-level aggregate noise, namely,

(67) $$Z_{u}^{Y}=\beta_{u}^{⊤}N=e_{1}^{⊤}D_{u}N.$$

With $B_{u}=\left[I_{K_{u}}\;\;{\tilde{B}}_{u}\right]\in F_{q}^{K_{u}\times V}$, where ${\tilde{B}}_{u}\in F_{q}^{K_{u}\times \left(V-K_{u}\right)}$. Choose

(68) $$R_{u}=\left[\begin{array}{c} I_{K_{u}}\\ L_{u} \end{array}\right],\;L_{u}\in F_{q}^{\left(V-K_{u}\right)\times K_{u}}.$$

Then

(69) $$B_{u}R_{u}=\left[I_{K_{u}}\;\;{\tilde{B}}_{u}\right]\left[\begin{array}{c} I_{K_{u}}\\ L_{u} \end{array}\right]=I_{K_{u}}+{\tilde{B}}_{u}L_{u}.$$

Since the right-hand side is a $K_{u}\times K_{u}$ square matrix, we have

(70) $$\mathrm{rank}\left(B_{u}R_{u}\right)=K_{u}\Leftrightarrow \det\;\left(I_{K_{u}}+{\tilde{B}}_{u}L_{u}\right)\neq 0.$$

Among all solutions of the linear constraint, we choose $L_{u}$ such that $I_{K_{u}}+{\tilde{B}}_{u}L_{u}$ is nonsingular; such a choice exists over a sufficiently large finite field $F_{q}$.

Using a common user-level encoding matrix $R_{u}$ for the *V* users in cluster *u*, define

(71) $$\left[\begin{array}{c} Z_{u,1}\\ \vdots \\ Z_{u,V} \end{array}\right]≜R_{u}N^{\left(u\right)}=R_{u}D_{u}N.$$

To ensure correctness after relay aggregation, we impose

(72) $$1_{V}^{⊤}\left[\begin{array}{c} Z_{u,1}\\ \vdots \\ Z_{u,V} \end{array}\right]=Z_{u}^{Y}=1_{V}^{⊤}R_{u}\;N^{\left(u\right)}.$$

From (67) and (72), it follows that:

(73) $$1_{V}^{⊤}R_{u}=e_{1}^{⊤}.$$

Substituting $R_{u}=\left[\begin{array}{c} I_{K_{u}}\\ L_{u} \end{array}\right]$ yields the equivalent condition

(74) $$1_{V-K_{u}}^{⊤}L_{u}=e_{1}^{⊤}-1_{K_{u}}^{⊤}.$$

Since rank$\left(R_{u}\right)=K_{u}$, the left null space of $R_{u}$ has dimension $V-K_{u}$. Thus there exists a full-row-rank $R_{u}^{⊥}\in F_{q}^{(V-K_{u})\times V}$ with $R_{u}^{⊥}R_{u}=0.$

Let us prove the above scheme is correct and secure. For correctness (refer to (8)), we have

(75) $$\begin{array}{cc} F=FS\; & \overset{\left(56\right)}{=}\left[S_{1};\ldots ;S_{M}\right]+\tilde{F}\left[S_{M+1};\ldots ;S_{U}\right]\\ & \overset{\left(60\right)}{=}\left[Y_{1};\ldots ;Y_{M}\right]+\tilde{F}\left[Y_{M+1};\ldots ;Y_{U}\right]. \end{array}$$

so that F can be decoded correctly from ${\left(Y_{u}\right)}_{u\in [U]}$.

For relay security (refer to (10)), we have

(76) $$\begin{array}{cc} & I(\left\{B_{u}\right\};{\left\{X_{u,v}\right\}}_{v\in [V]}) \end{array}$$

(77) $$\begin{array}{cc} & =H\left({\left\{X_{u,v}\right\}}_{v\in [V]}\right)-H\;\left({\left\{X_{u,v}\right\}}_{v\in [V]}|B_{u}\right) \end{array}$$

(78) $$\begin{array}{cc} & \leq V-H\;\left({\left\{X_{u,v}\right\}}_{v\in [V]},\;R_{u}^{⊥}\left[X_{u,1};\ldots ;X_{u,V}\right]|B_{u}\right) \end{array}$$

(79) $$\begin{array}{cc} & =V-H\;\left(R_{u}^{⊥}\left[W_{u,1};\ldots ;W_{u,V}\right]|B_{u}\right)-H\;\left({\left\{X_{u,v}\right\}}_{v\in [V]}|R_{u}^{⊥}\left[W_{u,1};\ldots ;W_{u,V}\right],\;B_{u}\right) \end{array}$$

(80) $$\begin{array}{cc} & =\;V-\left(V-K_{u}\right)-H\left(N^{\left(u\right)}\right) \end{array}$$

(81) $$\begin{array}{cc} & =V-V+K_{u}-K_{u}=0. \end{array}$$

In (79), $R_{u}^{⊥}R_{u}=0$ guarantees that the injected keys are zero-forced in $R_{u}^{⊥}\left[X_{u,1};\ldots ;X_{u,V}\right]$, and the last equality follows from $\mathrm{rank}\left(R_{u}^{⊥}\right)=V-K_{u}$ and $\mathrm{rank}(\left[B_{u};R_{u}^{⊥}\right])=V$.

For server security (refer to (11)), we have

(82) $$\begin{array}{cc} I\;\left(G;{\left(Y_{u}\right)}_{u\in [U]}|F\right) & =H\;\left({\left(Y_{u}\right)}_{u\in [U]}|F\right)-H\;\left({\left(Y_{u}\right)}_{u\in [U]}|G,F\right) \end{array}$$

(83) $$\begin{array}{cc} & =H\;\left({\left(Y_{u}\right)}_{u\in [U]},F\right)-H\left(F\right)-H\;\left({\left(Y_{u}\right)}_{u\in [U]},Q\left[Y_{M+1};\ldots ;Y_{U}\right]|G,F\right) \end{array}$$

(84) $$\begin{array}{cc} & \overset{\left(8)(60\right)}{=}H\;\left({\left(Y_{u}\right)}_{u\in [U]}\right)-H\left(F\right)-H\;\left({\left(Y_{u}\right)}_{u\in [U]},Q\left[S_{M+1};\ldots ;S_{U}\right]|G,F\right)\\ & \leq \left(U-M\right)-H\;\left(Q[S_{M+1};\ldots ;S_{U}]|G,F\right) \end{array}$$

(85) $$\begin{array}{cc} & \;-H\;\left({\left(Y_{u}\right)}_{u\in [U]}|Q\left[S_{M+1};\ldots ;S_{U}\right],G,F\right) \end{array}$$

(86) $$\begin{array}{cc} & \overset{\left(57\right)}{=}\left(U-M\right)-\left[U-\mathrm{rank}([F;G])\right]-H\;\left({\left(Y_{u}\right)}_{u\in [U]}|{\left(S_{u}\right)}_{u\in [U]}\right) \end{array}$$

(87) $$\begin{array}{cc} & \overset{\left(60\right)}{=}\mathrm{rank}\left(\left[F;G\right]\right)-M-H\;\left(T|{\left(S_{u}\right)}_{u\in [U]}\right) \end{array}$$

(88) $$\begin{array}{cc} & \overset{\left(60\right)}{=}\mathrm{rank}\left(\left[F;G\right]\right)-M-H\;\left(Q^{⊥}P|{\left(S_{u}\right)}_{u\in [U]}\right) \end{array}$$

(89) $$\begin{array}{cc} & =\mathrm{rank}\left(\left[F;G\right]\right)-M-H\;\left(P|{\left(S_{u}\right)}_{u\in [U]}\right) \end{array}$$

(90) $$\begin{array}{cc} & =\mathrm{rank}\left(\left[F;G\right]\right)-M-H\;\left(AN|{\left(S_{u}\right)}_{u\in [U]}\right) \end{array}$$

(91) $$\begin{array}{cc} & =\mathrm{rank}([F;G])-M-\mathrm{rank}(G|F) \end{array}$$

(92) $$\begin{array}{cc} & =\mathrm{rank}([F;G])-M-[\mathrm{rank}([F;G])-M]=0. \end{array}$$

Fundamentally, our design methodology reconstructs the solution by working backward from the security requirement in (11). The condition of vanishing mutual information implies that the conditional entropy $H({\left(Y_{u}\right)}_{u\in [U]}|G,F)$ must saturate the value U−M. We accomplish this by utilizing Q to isolate a signal-bearing subspace independent of channel realizations and maximizing its rank. Consequently, the keys injection is projected exclusively onto $Q^{⊥}$. This geometric arrangement ensures that the security threshold is satisfied with the minimum necessary keys dimensions.

## 6. Converse

We begin with a useful lemma. It states that each user message $X_{u,v}$ must contain at least *L* symbols of information, even when all other inputs are revealed. Similarly, each relay message $Y_{u}$ must carry at least *L* symbols whenever there exists at least one connected input $X_{u,v}$ that remains unknown.

**Lemma** **1.** *For any u∈[U],v∈[V], we have*

(93) $$\begin{array}{cc} H\;\left(X_{u,v}\;|\;{\left\{W_{i,j},Z_{i,j}\right\}}_{\left(i,j)\in [U]\times [V]\setminus \{(u,v)\right\}}\right) & \geq L, \end{array}$$

(94) $$\begin{array}{cc} H\;\left(Y_{u}\;|\;{\left\{W_{i,j},Z_{i,j}\right\}}_{\left(i,j)\in [U]\times [V]\setminus \{(u,v)\right\}}\right) & \geq L. \end{array}$$

**Proof.**  Consider (93), we have

$$\begin{array}{cc} & H\left(X_{u,v}|{\left\{W_{i,j},Z_{i,j}\right\}}_{\left(i,j)\in [U]\times [V]\setminus \{(u,v)\right\}}\right) \end{array}$$

(95) $$\begin{array}{cc} & \geq I\left(X_{u,v};F|{\left\{W_{i,j},Z_{i,j}\right\}}_{\left(i,j)\in [U]\times [V]\setminus \{(u,v)\right\}}\right)\\ & =H\left(F|{\left\{W_{i,j},Z_{i,j}\right\}}_{\left(i,j)\in [U]\times [V]\setminus \{(u,v)\right\}}\right) \end{array}$$

(96) $$\begin{array}{cc} & \;-H\left(F|X_{u,v},{\left\{W_{i,j},Z_{i,j}\right\}}_{\left(i,j)\in [U]\times [V]\setminus \{(u,v)\right\}}\right)\\ & \overset{\left(3),(4\right)}{=}H\left(W_{u,v}|{\left\{W_{i,j},Z_{i,j}\right\}}_{\left(i,j)\in [U]\times [V]\setminus \{(u,v)\right\}}\right) \end{array}$$

(97) $$\begin{array}{cc} & \;-\underset{\overset{\left(8\right)}{=}0}{\underset{︸}{H\left(F|X_{u,v},{\left\{W_{i,j},Z_{i,j}\right\}}_{\left(i,j)\in [U]\times [V]\setminus \{(u,v)\right\}},Y_{\left[U\right]}\right)}} \end{array}$$

(98) $$\begin{array}{cc} & \overset{\left(1\right)}{=}H\left(W_{u,v}\right)=L. \end{array}$$

where the last step is due to the independence of the inputs and the keys.

The proof of (94) is similar to that of (93):

$$\begin{array}{cc} & H\left(Y_{u}|{\left\{W_{i,j},Z_{i,j}\right\}}_{\left(i,j)\in [U]\times [V]\setminus \{(u,v)\right\}}\right) \end{array}$$

(99) $$\begin{array}{cc} & =I\left(Y_{u};F|{\left\{W_{i,j},Z_{i,j}\right\}}_{\left(i,j)\in [U]\times [V]\setminus \{(u,v)\right\}}\right)\\ & =H\left(F|{\left\{W_{i,j},Z_{i,j}\right\}}_{\left(i,j)\in [U]\times [V]\setminus \{(u,v)\right\}}\right) \end{array}$$

(100) $$\begin{array}{cc} & \;-\underset{\overset{\left(3),(4),(8\right)}{=}0}{\underset{︸}{H\left(F|Y_{u},{\left\{W_{i,j},Z_{i,j}\right\}}_{\left(i,j)\in [U]\times [V]\setminus \{(u,v)\right\}}\right)}} \end{array}$$

(101) $$\begin{array}{cc} & =H(W_{u,v})=L. \end{array}$$

Note that in the proof of (93) and (94), only the correctness constraint (8) is imposed and the security constraints (10) and (11) are not used.

**Lemma** **2.** *For any u∈[U], we demonstrate that the messages must not disclose excessive information regarding the inputs, as doing so would violate the security constraint* (10).

(102) $$\begin{array}{cc} \; & I\;\left({\left\{X_{u,v}\right\}}_{v\in [V]};{\left\{W_{u,v}\right\}}_{v\in [V]}\right)\leq \left(V-\mathrm{rank}(B_{u})\right)L.\forall u\in \left[U\right]. \end{array}$$

**Proof.**  $$\begin{array}{cc} \; & I\;\left({\left\{X_{u,v}\right\}}_{v\in [V]};{\left\{W_{u,v}\right\}}_{v\in [V]}\right) \end{array}$$

(103) $$\begin{array}{cc} \; & \leq I\;\left({\left\{X_{u,v}\right\}}_{v\in [V]};{\left\{W_{u,v}\right\}}_{v\in [V]},B_{u}\right) \end{array}$$

(104) $$\begin{array}{cc} \; & =\underset{\overset{\left(10\right)}{=}0}{\underset{︸}{I\;\left({\left\{X_{u,v}\right\}}_{v\in [V]};B_{u}\right)}}+I\;\left({\left\{X_{u,v}\right\}}_{v\in [V]};{\left\{W_{u,v}\right\}}_{v\in [V]}|B_{u}\right) \end{array}$$

(105) $$\begin{array}{cc} \; & \leq H\;\left({\left\{W_{u,v}\right\}}_{v\in [V]}|B_{u}\right) \end{array}$$

(106) $$\begin{array}{cc} \; & \leq H\;\left({\left\{W_{u,v}\right\}}_{v\in [V]},B_{u}\right)-H\;\left(B_{u}\right) \end{array}$$

(107) $$\begin{array}{cc} \; & =\left(V-\mathrm{rank}(B_{u})\right)L. \end{array}$$

The first term in (104) is zero due to the relay security constraint (10).

**Lemma** **3.** *Consider any G and F, the received signals must not reveal any information about the individual inputs beyond the aggregated result, as otherwise, the server security constraint* (11) *would be violated. we have*

(108) $$\begin{array}{c} I\left({\left\{Y_{u}\right\}}_{u\in [U]}\;;\;{\left\{S_{u}\right\}}_{u\in [U]}\right)\leq \left(U-\mathrm{rank}(G|F)\right)L. \end{array}$$

**Proof.**  (109) $$\begin{array}{cc} \; & I\left({\left\{Y_{u}\right\}}_{u\in [U]};{\left\{S_{u}\right\}}_{u\in [U]}\right) \end{array}$$

(110) $$\begin{array}{cc} \; & =I\left({\left\{Y_{u}\right\}}_{u\in [U]};{\left\{S_{u}\right\}}_{u\in [U]},G\right) \end{array}$$

(111) $$\begin{array}{cc} \; & =I\left({\left\{Y_{u}\right\}}_{u\in [U]};G\right)+I\left({\left\{Y_{u}\right\}}_{u\in [U]};{\left\{S_{u}\right\}}_{u\in [U]}\;|\;G\right) \end{array}$$

(112) $$\begin{array}{cc} \; & \leq I\left({\left\{Y_{u}\right\}}_{u\in [U]},F;G\right)+H\left({\left\{S_{u}\right\}}_{u\in [U]}\;|\;G\right) \end{array}$$

(113) $$\begin{array}{cc} \; & =I\left(F;G\right)+\underset{\overset{\left(11\right)}{=}0}{\underset{︸}{I\left(G;{\left\{Y_{u}\right\}}_{u\in [U]}\;|\;F\right)}}+H\left({\left\{S_{u}\right\}}_{u\in [U]},G\right)-H\left(G\right) \end{array}$$

(114) $$\begin{array}{cc} \; & =H\left(G\right)-H\left(G|F\right)+H\left({\left\{S_{u}\right\}}_{u\in [U]},G\right)-H\left(G\right) \end{array}$$

(115) $$\begin{array}{cc} \; & \leq (\mathrm{rank}(G)-\mathrm{rank}(G|F))L+(U-\mathrm{rank}(G))L \end{array}$$

(116) $$\begin{array}{cc} \; & =(U-\mathrm{rank}(G|F))L. \end{array}$$

The third term in (113) is zero due to the server security constraint (11).

**Proof** **of** $R_{Z_{\Sigma }}\geq \max\left\{\max_{u\in [U]}K_{u},\;\mathrm{rank}\left(G|F\right)\right\}.$ Building on the above lemmas, we complete the proof of the converse.

*First, we show that $R_{Z_{\Sigma }}\geq \max_{u\in [U]}K_{u}$.* By Lemma 2, we have

(117) $$\begin{array}{cc} L_{Z_{\Sigma }} & \geq H(Z_{\Sigma }) \end{array}$$

(118) $$\begin{array}{cc} \; & \geq H\left({\left\{Z_{u,v}\right\}}_{v\in [V]}\right) \end{array}$$

(119) $$\begin{array}{cc} \; & \geq I\left({\left\{Z_{u,v}\right\}}_{v\in [V]};{\left\{X_{u,v}\right\}}_{v\in [V]}\;|\;{\left\{W_{u,v}\right\}}_{v\in [V]}\right) \end{array}$$

(120) $$\begin{array}{cc} \; & =H\left({\left\{X_{u,v}\right\}}_{v\in [V]}\;|\;{\left\{W_{u,v}\right\}}_{v\in [V]}\right) \end{array}$$

(121) $$\begin{array}{cc} \; & =H\left({\left\{X_{u,v}\right\}}_{v\in [V]}\right)-I\left({\left\{X_{u,v}\right\}}_{v\in [V]};{\left\{W_{u,v}\right\}}_{v\in [V]}\right) \end{array}$$

(122) $$\begin{array}{cc} \; & \geq \sum_{v\in [V]}H\left(X_{u,v}\;|\;{\left\{W_{i,j},Z_{i,j}\right\}}_{\left(i,j)\in [U]\times [V]\setminus \{(u,v)\right\}}\right)-I\left({\left\{X_{u,v}\right\}}_{v\in [V]};{\left\{W_{u,v}\right\}}_{v\in [V]}\right) \end{array}$$

(123) $$\begin{array}{cc} \; & \overset{\left(93)(102\right)}{\geq }VL-\left(V-\mathrm{rank}(B_{u})\right)L=K_{u}L. \end{array}$$

Therefore,

(124) $$R_{Z_{\Sigma }}=\frac{L_{Z_{\Sigma }}}{L}\geq \max_{u\in [U]}K_{u}.$$

*Second, we show that $R_{Z_{\Sigma }}\geq \mathrm{rank}\left(G|F\right)$.* By Lemma 3, we have

(125) $$\begin{array}{cc} L_{Z_{\Sigma }} & \geq H(Z_{\Sigma }) \end{array}$$

(126) $$\begin{array}{cc} \; & \geq H\left(Z_{\left[U]\times [V\right]}\right) \end{array}$$

(127) $$\begin{array}{cc} \; & \geq I\left(Z_{\left[U]\times [V\right]};{\left\{Y_{u}\right\}}_{u\in [U]}\;|\;{\left\{S_{u}\right\}}_{u\in [U]}\right) \end{array}$$

(128) $$\begin{array}{cc} \; & =H\left({\left\{Y_{u}\right\}}_{u\in [U]}\;|\;{\left\{S_{u}\right\}}_{u\in [U]}\right)-\underset{\overset{\left(6\right)}{=}0}{\underset{︸}{H\left({\left\{Y_{u}\right\}}_{u\in [U]}\;|\;{\left\{S_{u}\right\}}_{u\in [U]},Z_{\left[U]\times [V\right]}\right)}} \end{array}$$

(129) $$\begin{array}{cc} \; & =H\left({\left\{Y_{u}\right\}}_{u\in [U]}\right)-I\left({\left\{S_{u}\right\}}_{u\in [U]};{\left\{Y_{u}\right\}}_{u\in [U]}\right) \end{array}$$

(130) $$\begin{array}{cc} \; & \geq \sum_{u=1}^{U}H\left(Y_{u}\;|\;{\left\{W_{i,j},Z_{i,j}\right\}}_{\left(i,j)\in [U]\times [V]\setminus \{(u,j):j\in [V]\right\}}\right)-I\left({\left\{S_{u}\right\}}_{u\in [U]};{\left\{Y_{u}\right\}}_{u\in [U]}\right) \end{array}$$

(131) $$\begin{array}{cc} \; & \overset{\left(94)(108\right)}{\geq }UL-\left(U-\mathrm{rank}(G|F)\right)L \end{array}$$

(132) $$\begin{array}{cc} \; & =\mathrm{rank}(G|F)\;L. \end{array}$$

Hence,

(133) $$R_{Z_{\Sigma }}=\frac{L_{Z_{\Sigma }}}{L}\geq \mathrm{rank}\left(G|F\right).$$

Combining (124) and (133), we obtain

$$R_{Z_{\Sigma }}\geq \max\left\{\max_{u\in [U]}K_{u},\;\mathrm{rank}\left(G|F\right)\right\}.$$

## 7. Conclusions

This paper investigates information theoretic secure aggregation of linear functions over a two hop hierarchical network with relay-assisted communication. By jointly accounting for relay-level privacy constraints and server-side function-specific security requirements, we establish a unified framework for hierarchical vector linear secure aggregation.

Our main contribution is a complete characterization of the optimal communication key rate region. We show that both hops achieve the minimum possible communication rate of one symbol per input symbol, while the required source key rate is governed by the maximum of the intra-cluster security requirement and the conditional rank rank(G∣F). This result demonstrates that hierarchical architectures incur no additional communication cost compared to single hop systems, while substantially reducing the masking burden at the server through structured key injection.

To achieve these fundamental limits, we propose an explicit linear coding scheme based on systematic precoding, subspace alignment, and zero forcing. The scheme exploits the algebraic structure of the authorized and unauthorized functions to inject randomness exclusively into dimensions that do not interfere with the authorized computation. The achievability and converse proofs together establish that the derived rate region is information theoretically tight.

Overall, this work clarifies the fundamental role of hierarchy in secure aggregation and provides theoretical guidance for the design of scalable privacy preserving distributed learning systems. Future work includes extending the framework to scenarios with collusion among servers, relays, and users, as well as investigating robustness under user dropouts, heterogeneous cluster sizes, and asymmetric communication constraints.

## Figures and Tables

**Table 1:** Comparison between representative single-hop vector linear schemes and the proposed two-hop hierarchical scheme.

Aspect	Representative Single-Hop Vector Linear Schemes	Proposed Two-Hop Hierarchical Scheme
Architecture	Single-hop	Two-hop hierarchical
Trust model	Honest-but-curious server	Semi-trusted relays and honest-but-curious server
Security target	Server-side target-function privacy	Relay-side $B_{u}$-function privacy and server-side target-function privacy
Communication efficiency	Optimal communication rate: $R_{X}=1$	Optimal communication rates on both hops: $R_{X}=1,\;R_{Y}=1$
Technical challenge	Single-layer code design	Unified linear design under coupled relay/server security constraints

## Data Availability

No new data were created or analyzed in this study. Data sharing is not applicable.
